# Case Report: Non-traumatic splenic rupture as the first presentation of diffuse large B-cell lymphoma complicated by clinically diagnosed pulmonary aspergillosis supported by metagenomic next-generation sequencing

**DOI:** 10.3389/fmed.2026.1867881

**Published:** 2026-08-13

**Authors:** Yuyue Zhao, Jun Duan

**Affiliations:** Department of Critical Care Medicine, China-Japan Friendship Hospital, Beijing, China

**Keywords:** clinically diagnosed pulmonary aspergillosis, diffuse large B-cell lymphoma, immunocompromised host, metagenomic next-generation sequencing, non-traumatic splenic rupture, splenectomy

## Abstract

Non-traumatic splenic rupture is an uncommon, life-threatening event and may be the first sign of an unrecognized hematological malignancy. We describe a 60-year-old man who presented with hemorrhagic shock due to splenic rupture. Emergency splenectomy controlled the bleeding and provided the diagnostic specimen. Histology supported diffuse large B-cell lymphoma, not otherwise specified (DLBCL, NOS), with a non-germinal-center B-cell phenotype by the Hans algorithm and a Ki-67 index of approximately 90%. Epstein-Barr virus-encoded RNA was negative. Because fluorescence *in situ* hybridization for MYC, BCL2, and BCL6 rearrangements was unavailable, high-grade B-cell lymphoma with rearrangements could not be excluded. Staging was incomplete; the case is therefore described as DLBCL with dominant splenic presentation rather than primary splenic DLBCL. The post-operative course was complicated by severe pneumonia. Pulmonary aspergillosis was clinically diagnosed based on the overall host profile, bronchoscopic findings, serum galactomannan positivity, BALF mNGS results, and subsequent culture. BALF mNGS detected Aspergillus fumigatus and Pseudomonas aeruginosa before culture confirmation. Voriconazole was started, but the patient died on post-operative day 11, about 24 h later. Splenic rupture was therefore the first manifestation of DLBCL, and BALF mNGS served as supportive, rather than standalone, evidence for pulmonary aspergillosis in a critically ill immunocompromised patient.

## 1 Introduction

Non-traumatic splenic rupture is encountered infrequently in clinical practice, and when it occurs the initial presentation is often indistinguishable from other causes of acute hemoperitoneum. Delay in controlling hemorrhagic shock raises the risk of irreversible hemodynamic compromise, which makes prompt recognition essential (1). Among the established etiologies, hematological malignancies account for about 14% of cases in the largest available systematic review, and non-Hodgkin lymphoma (NHL) represents the single most common hematological cause (1). DLBCL constitutes roughly 30% of NHL diagnoses and is the most common aggressive NHL subtype; although the absolute number of reported DLBCL-associated ruptures remains small, its rapid growth kinetics and frequent extranodal involvement make it clinically important in this setting (2).

What makes DLBCL-related rupture diagnostically challenging is the frequent absence of the features clinicians usually rely upon to suspect lymphoma. The spleen may be the predominant clinically apparent site, with no peripheral lymphadenopathy and no FDG-avid lesions on PET-CT, a scenario reported in approximately 3% of DLBCL cases (3). This presentation is rare because DLBCL is usually nodal or disseminated at diagnosis; isolated or dominant splenic disease can remain clinically silent until rapid tumor expansion, necrosis, vascular compromise, thrombocytopenia, or capsular tension causes hemorrhage.

We report a patient in whom emergency splenectomy was both life-saving and the first diagnostic intervention for DLBCL. Severe post-operative pneumonia followed, with pulmonary aspergillosis diagnosed clinically rather than by strict EORTC/MSGERC probable-disease criteria. Susceptibility was likely multifactorial: lymphoma-associated immune dysfunction provided the underlying substrate, compounded by critical illness, with mechanical ventilation, broad-spectrum antibacterial exposure, emphysema, and recent surgery among the contributing factors. Splenectomy played a contributing rather than primary role. The case also illustrates the supportive role and limitations of BALF mNGS in a critically ill hematology patient.

## 2 Case description

### 2.1 Patient background

A 60-year-old male presented with 3 weeks of intermittent exertional dyspnea and 6 days of worsening abdominal pain. He had been treated for hypertension and a prior cerebrovascular infarction for over a decade and was taking antihypertensive agents and low-dose aspirin (100 mg/day). He had smoked approximately 10 cigarettes daily for 20 years. There was no history of trauma, no personal or family history of lymphoma or other hematological malignancy, and no recent overseas travel or animal contact.

### 2.2 Initial clinical findings

Dyspnea began 22 days before admission, limiting his walking distance to roughly 10 meters before he had to stop, and was accompanied by bilateral leg edema and reduced urine output. Eight days prior to admission, abdominal pain developed and worsened progressively over the following 8 days. Pre-admission blood tests (1 day before presentation) showed Hb 101 g/L, PLT 67 × 10^9^/L, AST 147 IU/L, total bilirubin 75.25 μmol/L, direct bilirubin 47.54 μmol/L, CRP 58 mg/L, procalcitonin 2.7 ng/mL, and reticulocytes 140 × 10^9^/L (5.7%). Peripheral blood smear examination showed band neutrophils 2%, segmented neutrophils 96%, and lymphocytes 2%, indicating marked neutrophilic predominance with severe relative lymphopenia. The smear also showed increased white blood cells with neutrophil hypersegmentation and vacuolar degeneration, anisocytosis with occasional macrocytes, and markedly decreased platelets with large platelets. No nucleated red blood cells or hemoparasites were detected. These abnormalities remained unexplained until the patient's emergency admission.

On arrival the patient was diaphoretic and hemodynamically unstable (heart rate 118 bpm, blood pressure 88/52 mmHg). The abdomen was distended and diffusely tender. Peripheral lymphadenopathy was not identified, and the spleen was not clinically palpable on initial examination; there were no features to suggest underlying chronic liver disease. Hemoglobin had fallen from 101 to 64 g/L, and arterial lactate was 4.3 mmol/L against a background of mild metabolic acidosis (pH 7.43). Abdominal CT demonstrated perisplenic fluid with no intra-abdominal lymphadenopathy, and diagnostic paracentesis of the right lower quadrant yielded non-coagulating blood. This clinical picture established splenic rupture with hemorrhagic shock as the working diagnosis.

### 2.3 Emergency surgery

Emergency exploratory laparotomy with splenectomy was performed. Intraoperative findings included a markedly enlarged spleen with a 5-cm full-thickness laceration on its posterior surface, active arterial hemorrhage, and extensive perisplenic blood clots. Fresh frozen plasma (800 mL) and one platelet unit were transfused intraoperatively; estimated blood loss was approximately 300 mL. Norepinephrine infusion (0.4 μg/kg/min) was required throughout, and the patient was transferred to the ICU intubated following the procedure.

### 2.4 Histopathological diagnosis

**Figure 1:**
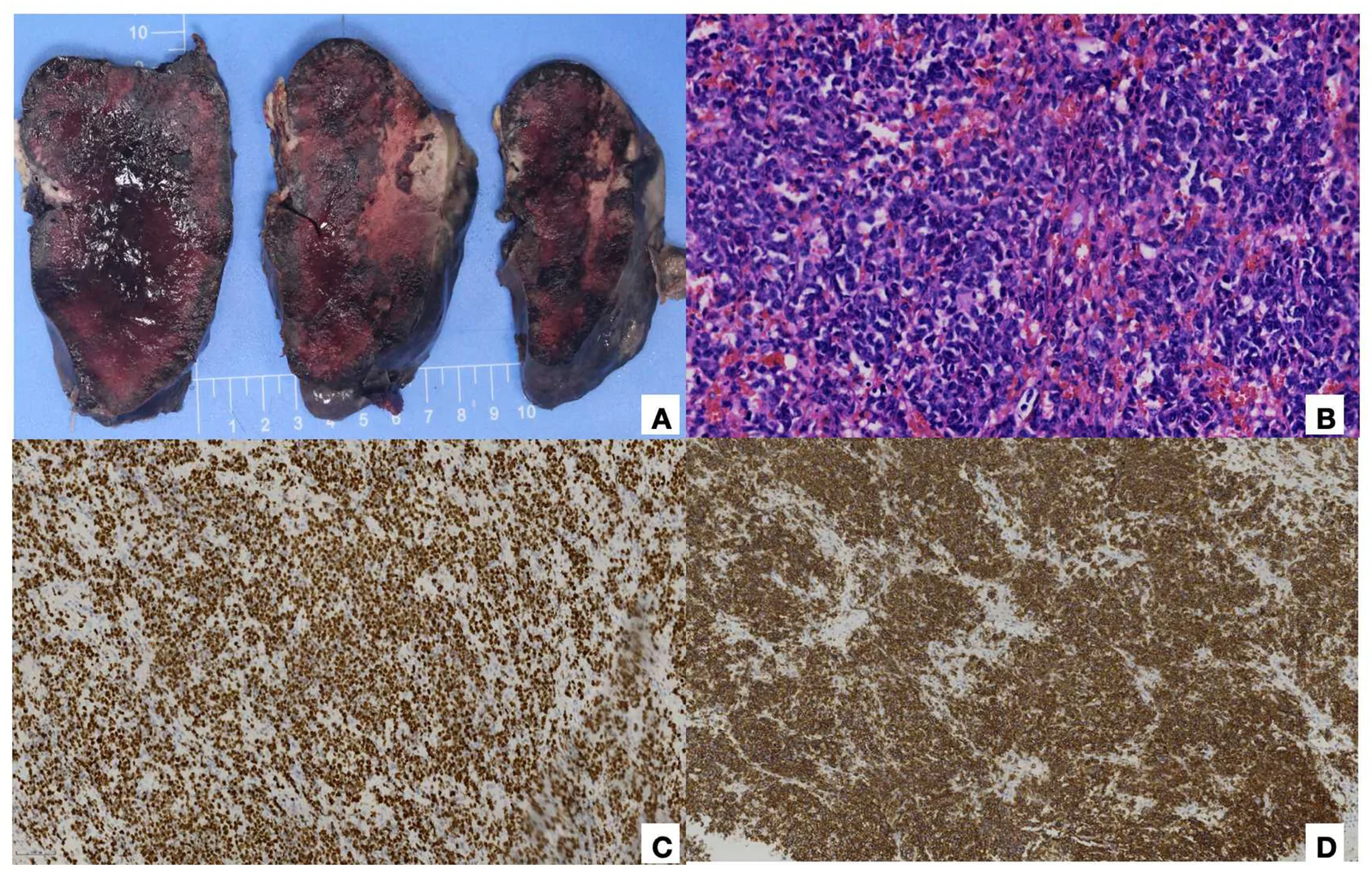
Histopathological and immunohistochemical features of the resected spleen. **(A)** Intraoperative appearance showing splenomegaly and posterior capsular rupture. **(B)** Hematoxylin and eosin staining (×200): diffuse large lymphoid cell infiltration with necrosis and hemorrhage. **(C)** CD20 immunohistochemistry (×100): strong diffuse positivity in neoplastic cells. **(D)** Ki-67 immunohistochemistry (×100): proliferation index ~90%.

The resected spleen showed diffuse infiltration by large atypical lymphoid cells with irregular nuclei, vesicular chromatin, and prominent nucleoli, together with extensive necrosis, hemorrhage, tumor thrombi, and perineural invasion. Immunohistochemistry demonstrated CD20+, BCL2+, BCL6+, MUM1+, Ki-67 ~90%; negative staining for CD3, CD10, CD21, and c-MYC (< 10%); and negative EBER *in situ* hybridization. Under the 5th edition WHO Classification of Haematolymphoid Tumors and the 2022 International Consensus Classification, the available findings supported diffuse large B-cell lymphoma, not otherwise specified (DLBCL, NOS), with a non-GCB phenotype assigned by the Hans algorithm (4–6). Low c-MYC immunohistochemical expression does not substitute for FISH. Because FISH for MYC, BCL2, and BCL6 rearrangements was unavailable, high-grade B-cell lymphoma with rearrangements could not be excluded. The family declined bone marrow biopsy, PET-CT, molecular studies, and further oncological work-up; accordingly, we describe DLBCL with dominant splenic presentation and do not infer primary splenic disease. The representative gross, histological, and immunohistochemical findings are shown in Figure 1.

### 2.5 Post-operative ICU course and infection diagnosis

During the early ICU course, acute-on-chronic respiratory failure developed in the context of pre-existing emphysema, requiring pressure-controlled mechanical ventilation (PaO_2_/FiO_2_ ratio was 113). Immunological assessment on post-operative day 1 revealed extensive deficits: IgG 454 mg/dL, IgM 48 mg/dL, C3 43.9 mg/dL, C4 13.9 mg/dL, CD4^+^ count 320/μL, CD8^+^ count 43/μL, and IL-6 3,624 pg/mL. LDH rose from 1,749 to 1,906 U/L over the first week. Empirical therapy with meropenem and vancomycin was started for presumed nosocomial pneumonia.

On post-operative day 9 (POD 9), the patient developed a temperature of 39.8 °C with rapidly worsening oxygenation. Chest radiography showed bilateral infiltrates, and bronchoscopy revealed abundant mucopurulent secretions and multiple white mucosal nodules. High-resolution CT was not performed post-operatively due to critical instability, so classic CT features of invasive pulmonary aspergillosis could not be assessed. Bronchoalveolar lavage fluid (BALF) was submitted for clinical metagenomic next-generation sequencing (mNGS), and DNA sequencing was performed on the Illumina NextSeq 550Dx platform. The report was issued about 24 h after bronchoscopy and identified *A. fumigatus* (2,302 reads; 93.12% relative abundance within fungi; 0.64% genome coverage) and *P. aeruginosa* (2,390,050 reads). The archived report classified both as detected pathogens but did not provide the assay-wide positivity threshold or negative/background-control results. Serum galactomannan was strongly positive (index 10.74), whereas 1,3-beta-D-glucan was negative. The patient's host factors, bronchoscopic findings, galactomannan results, mNGS findings, and subsequent fungal culture collectively supported a clinical diagnosis of pulmonary aspergillosis. Nevertheless, the case could not be classified as probable invasive pulmonary aspergillosis according to the EORTC/MSGERC criteria because the characteristic chest CT findings required by the criteria were absent (7). Intravenous voriconazole was started on POD 10, and vancomycin was discontinued.

#### 2.5.1 Metagenomic next-generation sequencing

BALF collected during bronchoscopy was submitted for mNGS. Total DNA was extracted from BALF specimens and sequencing libraries were prepared according to the manufacturer's validated clinical protocol. Sequencing was performed on the Illumina NextSeq 550Dx platform. After sequencing, low-quality, low-complexity, and short reads were removed, followed by subtraction of human-derived sequences through alignment to the human reference genome (hg38). The remaining high-quality microbial reads were aligned against a curated reference database containing bacterial, fungal, viral, and parasitic genomes for taxonomic classification.

Microorganism reporting was based on an integrated laboratory interpretation algorithm rather than raw read counts alone. The algorithm incorporated pathogen category, species-specific read counts, relative abundance, genomic coverage, organism ranking within each microbial class, comparison with negative or background controls, and known environmental contaminants. Sequencing results were interpreted alongside the patient's clinical manifestations and conventional microbiological findings. The laboratory reported results within about 24 h of specimen receipt.

### 2.6 Outcome

The patient's condition continued to deteriorate after voriconazole was started, but the roughly 24-h interval before death was too short to assess treatment response. After disclosure of the DLBCL diagnosis, the family declined further diagnostic procedures and treatment; formal staging, molecular studies, and chemotherapy were not pursued. The patient died on POD11 from refractory septic shock associated with severe pneumonia. Clinically diagnosed pulmonary aspergillosis and *P. aeruginosa* infection were considered contributory. A sputum culture obtained before death was finalized on POD14 and grew *A. fumigatus and P. aeruginosa*.

**Figure 2:**
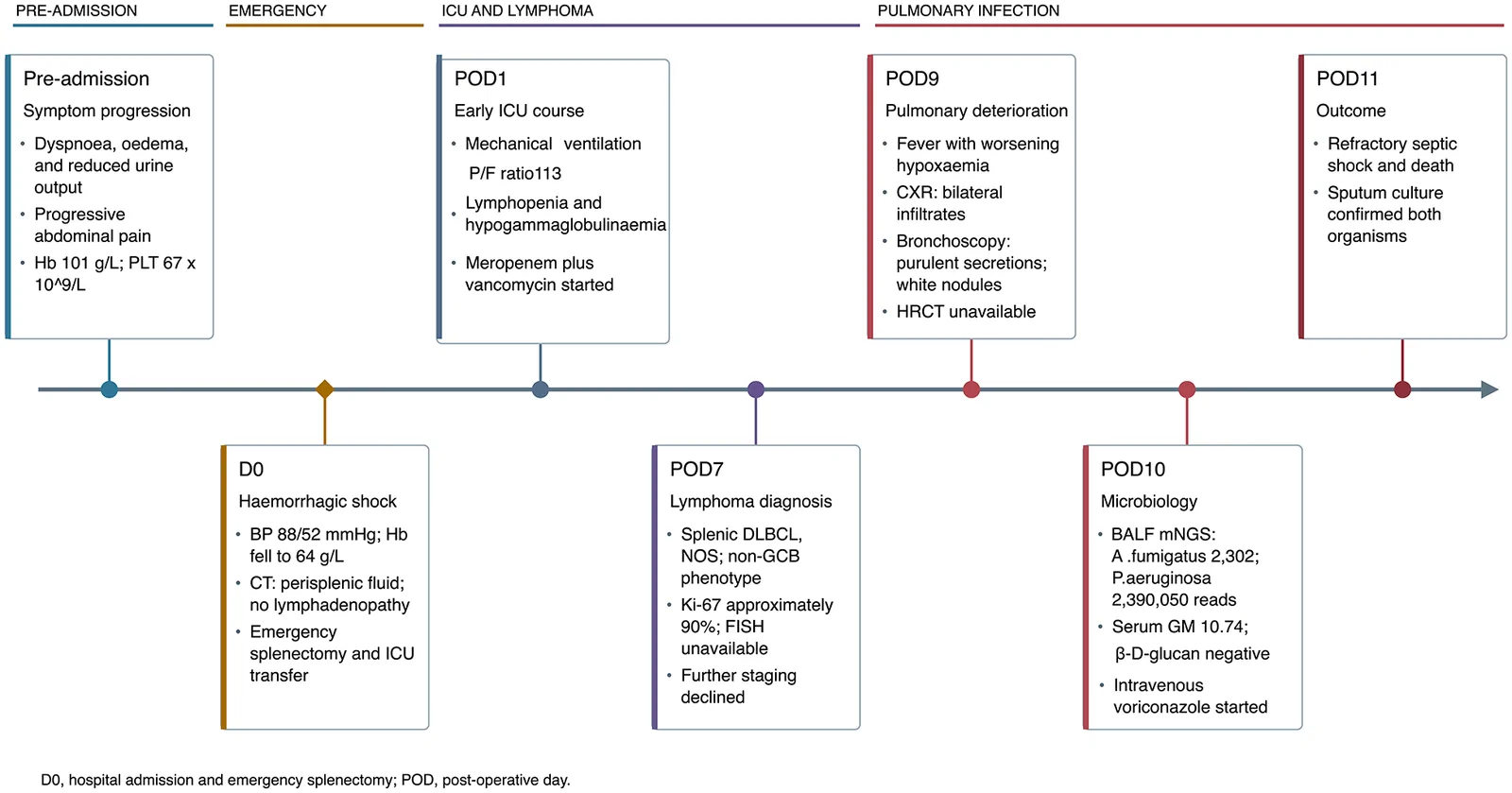
Relative clinical timeline from symptom onset to death. D0 indicates the day of emergency splenectomy. POD, postoperative day; BALF, bronchoalveolar lavage fluid; GM, galactomannan; mNGS, metagenomic next-generation sequencing.

A detailed chronological summary of clinical events, key laboratory values, and therapeutic interventions is presented in Figure 2.

## 3 Discussion

Among hematological malignancies associated with non-traumatic splenic rupture, DLBCL is reported most frequently, although the diagnosis is rarely established before the acute event. A systematic literature search identified five comparable cases published between 2018 and 2025 (Table 1) (8–12). The present case adds a sixth report.

**Table 1:** Published cases of non-traumatic splenic rupture (NTSR) attributable to diffuse large B-cell lymphoma (DLBCL), 2018–2025.

References	Age/sex	Clinical presentation	NTSR as first DLBCL presentation	Management	Outcome
Kaniappan et al. (8)	40 M	1-week LUQ pain + splenomegaly; no prior diagnosis. LDH elevated; CT: 18 × 15 × 16.9 cm hematoma.	Yes	Emergency splenectomy	Referred for R-CHOP; lost to follow-up.
Dunphy et al. (9)	61 M	Acute abdomen + hypovolemic shock (BP 90/57 mmHg); Kehr's sign; on warfarin. CT: 7.4-cm splenic mass + subcapsular hematoma.	Yes	Conservative → enlarged hematoma → splenic artery embolisation + biopsy	DLBCL confirmed; R-CHOP commenced.
Mori et al. (10)	60 M	Undiagnosed transverse myelitis 2 months prior; presyncope + preshock; disseminated lymphadenopathy.	Yes—transverse myelitis likely lymphoma-related	Attempted TAE failed → emergency laparotomy	Died: MOF + hemorrhage. Autopsy confirmed systemic DLBCL.
Guo et al. (11)	48 M	2-year FUO; elevated LDH + ferritin; splenomegaly; PET-CT NEGATIVE; bone marrow × 3 normal → hemorrhagic shock.	Yes—delayed by false-negative PET-CT	Emergency splenectomy	Zanubrutinib maintenance; clinically stable at follow-up.
Hu et al. (12)	N/R	Neck and shoulder pain only; no typical lymphoma features. FAST/CT confirmed splenic rupture.	Yes	Emergency splenectomy	Good recovery; primary splenic DLBCL confirmed.
Present case, 2026	60 M	21-day dyspnea + 6-day abdominal pain; long-term aspirin; hemorrhagic shock; Hb 101 → 64 g/L; LDH 1,749–1,906 U/L. Post-op: clinically diagnosed pulmonary aspergillosis plus *P. aeruginosa* supported by mNGS and culture	Yes	Emergency splenectomy; IV voriconazole + meropenem	Died post-op day 11: refractory septic shock with severe pneumonia; *A. fumigatus* and *P. aeruginosa* detected.

non-GCB, non-germinal center B-cell; FUO, fever of unknown origin; PET-CT, positron emission tomography–computed tomography; R-CHOP, rituximab-cyclophosphamide-doxorubicin-vincristine-prednisolone; MOF, multiple organ failure; TAE, transcatheter arterial embolisation; mNGS, metagenomic next-generation sequencing; IPA, invasive pulmonary aspergillosis.

Across the six cases, rupture was the first clinical manifestation and lymphoma had not been diagnosed beforehand. A related mantle cell lymphoma report shows that occult splenic lymphoma may also worsen the consequences of minor trauma (13). Splenic infiltration and enlargement likely set the stage for rupture, with tumor necrosis, vascular involvement, hemorrhage, thrombocytopenia, and coagulopathy acting as precipitants rather than independent causes. Our specimen showed necrosis, hemorrhage, and tumor thrombi, but not documented infarction. Before admission, cytopenias, hyperbilirubinemia, elevated AST, reticulocytosis, and raised inflammatory markers suggested an occult systemic disorder, although the underlying DLBCL was not recognized at that stage. Aspirin probably reduced the hemostatic reserve in a patient with thrombocytopenia and a structurally fragile spleen; it should not be presented as the cause of rupture.

Guo et al. described 2 years of fever, high LDH, splenomegaly, three negative marrow biopsies, and a non-avid PET-CT before rupture led to diagnosis (11). Non-FDG-avid DLBCL was reported in approximately 3% of DLBCL patients (6/222) within a cohort of 766 lymphoma patients (3). Thus, a negative PET-CT does not exclude splenic lymphoma when clinical suspicion remains high. In selected stable patients, image-guided biopsy or close surveillance may be preferable to unstructured observation.

Emergency splenectomy remains the appropriate life-saving and diagnostic procedure for hemodynamically unstable patients; all cases in Table 1 were diagnosed from splenic tissue. FAST can rapidly detect intraperitoneal fluid, whereas contrast-enhanced CT, when feasible, better defines splenic injury, hematoma, and lymphadenopathy (9). In stable patients, splenic artery embolisation may control bleeding and allow core biopsy, although subsequent splenectomy may still be required (9). Adequate tissue is essential for lymphoma classification and molecular risk assessment.

Although splenectomy is well known to impair clearance of encapsulated bacteria and T-cell-independent antibody responses, thereby underlying overwhelming post-splenectomy infection, this mechanism did not fully explain the infectious phenotype in the present patient (14, 15).

Aspergillus is not a classic post-splenectomy pathogen, and splenectomy alone is not a recognized major risk factor for pulmonary aspergillosis. The more plausible explanation lies in the cumulative burden of DLBCL-related immunosuppression—marked lymphopenia (CD4+ 320/μL; CD8+ 43/μL), hypogammaglobulinemia, and hypocomplementemia—layered onto critical illness, with mechanical ventilation, broad-spectrum antibacterial therapy, emphysema, and recent major surgery among the contributing factors. Splenectomy may have added to the overall immunological deficit but was unlikely to have been the principal cause.

Guidelines recommend vaccination against *S. pneumoniae, H. influenzae* type b, and *N. meningitidis*, with antibiotic prophylaxis considered for high-risk patients according to local policy (15). The emergency presentation and rapid deterioration precluded these measures, but planning should begin early after emergency splenectomy.

BALF mNGS provided an early microbiological signal rather than a definitive diagnosis. It reported *A. fumigatus* at 2,302 reads, with 93.12% relative abundance within fungi and 0.64% genome coverage, about 24 h after bronchoscopy and several days before culture confirmation. As Jiang et al. note, read counts alone can mislead; rank, relative abundance, and contamination-adjusted metrics carry more diagnostic weight (16), a caveat directly relevant to this report because the archived report did not include the assay-wide positivity threshold or negative/background-control results. Here, the result was supported by marked immune compromise, abnormal bronchoscopy, serum GM 10.74, and later recovery of *A. fumigatus* in culture. A negative beta-D-glucan did not exclude disease and is not Aspergillus-specific (7). Conversely, the absence of post-operative HRCT prevented assessment of the imaging component required for strict EORTC/MSGERC probable pulmonary aspergillosis (7). We therefore use the term clinically diagnosed pulmonary aspergillosis and regard mNGS as supportive evidence.

## 4 Conclusion

The main limitations are incomplete lymphoma staging and incomplete assay documentation. Because FISH for MYC, BCL2, and BCL6 was unavailable and the family declined PET-CT, bone marrow biopsy, and further molecular studies, high-grade B-cell lymphoma with rearrangements could not be excluded, and primary splenic DLBCL cannot be distinguished from systemic disease with dominant splenic presentation; cell-of-origin assignment likewise relied on the Hans algorithm rather than gene-expression profiling (6). Post-operative HRCT was not feasible, preventing assessment of imaging criteria for probable pulmonary aspergillosis. Although the mNGS platform and nucleic-acid workflow are described, the archived report did not contain assay-wide thresholds or negative/background-control results, which limits retrospective interpretation. Finally, the pre-admission laboratory abnormalities could not be fully interpreted because hemolysis and hepatobiliary investigations were incomplete.

For clinicians managing DLBCL after emergency splenectomy, absent lymphadenopathy or non-FDG-avid disease should not lower suspicion for splenic involvement when cytopenias, splenomegaly, hemorrhage, or unexplained inflammatory abnormalities are present. For mNGS results returned without assay-wide thresholds or control data, integration with radiological, bronchoscopic, microbiological, and mycological findings remains necessary before treatment decisions are finalized.

## Data Availability

The raw data supporting the conclusions of this article will be made available by the authors, without undue reservation.
